# Bulliform Phytolith Size of Rice and Its Correlation With Hydrothermal Environment: A Preliminary Morphological Study on Species in Southern China

**DOI:** 10.3389/fpls.2019.01037

**Published:** 2019-08-22

**Authors:** Can Wang, Houyuan Lu, Jianping Zhang, Limi Mao, Yong Ge

**Affiliations:** ^1^Department of Archaeology, School of History and Culture, Shandong University, Jinan, China; ^2^Key Laboratory of Cenozoic Geology and Environment, Institute of Geology and Geophysics, Chinese Academy of Sciences, Beijing, China; ^3^Center for Excellence in Tibetan Plateau Earth Science, Chinese Academy of Sciences, Beijing, China; ^4^College of Earth and Planetary Sciences, University of Chinese Academy of Sciences, Beijing, China; ^5^Nanjing Institute of Geology and Palaeontology, Chinese Academy of Sciences, Nanjing, China; ^6^Key Laboratory of Vertebrate Evolution and Human Origins, Institute of Vertebrate Paleontology and Paleoanthropology, Chinese Academy of Sciences, Beijing, China; ^7^Center for Excellence in Life and Paleoenvironment, Chinese Academy of Sciences, Beijing, China

**Keywords:** rice, bulliform phytolith, *Oryza sativa*, *Oryza rufipogon*, domestication, morphometric analysis

## Abstract

In the last decade, our understanding of rice domestication has improved by new archaeological findings using advanced analytical techniques such as morphological and morphometric analyses on rice grains, spikelet bases and phytoliths, and ancient DNA analysis on rice remains. Previous studies have considered the size of rice bulliform phytoliths as a proxy for tracking the domestication process. These phytoliths are often abundant and well preserved in sediments, and their shape is under the control of numerous genes, which may shift toward larger sizes by genetic mutation in domestication. Therefore, it has been assumed that the bulliforms of domesticated rice are usually larger than those of wild ones; however, morphometric data supporting this assumption are lacking in the literature, thereby requiring additional evidence to test its veracity. In this study, the vertical and horizonal lengths of bulliform phytoliths were measured in four rice species (domesticated *Oryza sativa* and wild *Oryza rufipogon*, *Oryza officinalis*, and *Oryza meyeriana*) from different regions of southern China. We found that the bulliform morphometric data of wild and domesticated rice overlapped and that there was no statistically significant difference between them. Therefore, bulliform size could not be used as a diagnostic indicator to distinguish domesticated rice from wild species and is a supporting rather than conclusive proxy for determining the domesticated status of rice in archaeological research. We further found that larger rice bulliform sizes likely occurred at the locations with higher temperature, precipitation, and water levels, indicating hydrothermal environment is an alternative factor influencing the size of rice bulliform phytoliths. For further archaeological use of an increasing size trend of bulliform phytoliths to reveal the process of rice domestication, we present some suggestions for controlling the influence of hydrothermal factors. Even so, the combination of bulliform phytolith size with other established criteria is strongly suggested to provide precise identification of wild and domesticated rice in future research.

## Introduction

Asian rice (*Oryza sativa* L.) is one of the most important crops and forms a staple food for more than half of the global population (Nayar, 2014). Understanding its origins and domestication from wild rice (*Oryza rufipogon* Griff.) is thus an important aim for researchers. There are two major subspecies of domesticated *O. sativa*, *Oryza sativa indica*, which is thought to have originated in the Himalayan region, and *Oryza sativa japonica*, which is thought to have originated in China (Londo et al., 2006). *Oryza rufipogon* is generally recognized as the ancestor of *Oryza sativa* (Wei et al., 2012), with two distinct domestication events leading to the two subspecies of domesticated rice (Londo et al., 2006). However, this hypothesis is still being vigorously debated as other evidence supports a single origin of Asian rice (Huang et al., 2012). There are more than 20 wild rice species recognized in the genus *Oryza* (Nayar, 2014; Stein et al., 2018), which belongs to the family: Poaceae and tribe: Oryzeae. Three of these species are found in China, *Oryza rufipogon*, *Oryza officinalis*, and *Oryza meyeriana* (Fan et al., 2000).

In the last decade, scientific understanding of rice domestication has greatly improved by new archaeological and genetic evidence using advanced analytical techniques such as flotation combined with morphometric analysis on rice grains and spikelet bases (Liu et al., 2007; Fuller et al., 2009; Gross and Zhao, 2014; Zheng et al., 2016), phytolith analysis (Wu et al., 2014; Huan et al., 2015; Zuo et al., 2017), pan-genome analysis (Wang et al., 2018a), and genome-wide association studies (Huang et al., 2012; Civáň and Brown, 2017; Choi and Purugganan, 2018). According to recent findings, a general consensus among scholars has been reached that rice was first domesticated in the middle and lower Yangtze River regions of southern China (Molina et al., 2011; Gross and Zhao, 2014; Silva et al., 2015; Choi et al., 2017; Zuo et al., 2017), although some consider the Pearl River region to also be a part of the original area where rice domestication occurred (Huang et al., 2012; Wei et al., 2012).

The estimated time of origin of rice domestication is before 13,000 BP, based on molecular clock analysis (Molina et al., 2011; Choi et al., 2017; Choi and Purugganan, 2018), which is much older than the earliest archaeological date of domestication (<10,000 BP) (Fuller et al., 2014; Larson et al., 2014). This disagreement may result from genetic studies that mainly focused on identifying the origins of alleles associated with domestication (e.g., *sh4*, *rc*, *laba1*, *prog1*), which likely emerged in wild rice prior to domestication (Choi et al., 2017; Civáň and Brown, 2017). On the other hand, archaeological studies attempted to detect the first appearance of morphological traits associated with domestication in archaeobotanical remains (Fuller et al., 2009; Jones and Liu, 2009). The molecular and archaeological chronologies may date two main phases in the macroevolutionary process: the emergence of a trait and the success of that trait (the trait becomes quantitatively significant within a population) (Katz, 2019). However, the most notable chronological dispute over the rice domestication is between two archaeological opinions: one suggests that the process of rice domestication may have begun around 10,000–9,000 BP (Liu et al., 2007; Zheng et al., 2007; Wu et al., 2014; Zheng et al., 2016; Zuo et al., 2017), while the other suggests that domestication of rice did not occur until around 8,000–6,000 BP (Fuller et al., 2007; Fuller et al., 2008; Fuller et al., 2009; Fuller et al., 2010; Larson et al., 2014). This debate is largely attributable to the differences in the methods employed, and the criteria used by various authors to identify domestication in rice remain from early sites in the Yangtze River region such as Shangshan, Kuahuqiao, and Xiaohuangshan. Establishing accurate and feasible criteria for distinguishing between domesticated and wild rice is thus of prime importance.

Three important lines of archaeological evidence have frequently been used in China, including grain size and morphological characteristics, spikelet bases, and phytoliths (Liu and Chen, 2012; Fuller and Castillo, 2014; Fuller, 2018). Grain morphometrics are considered to be semi-domestication traits and may not be diagnostic indicators of early domestication (Fuller, 2007; Fuller et al., 2007), mostly due to the considerable variation and overlap in length between domesticated and wild populations which leads to some proportion of false assignments in ancient rice grains (Fuller, 2007). Many studies have focused on the form of spikelet base, which is thought to be the most diagnostic trait in rice remains in terms of identifying domestication status (Fuller, 2018). However, these are insufficient, because the distinctive characteristics of immaturity, shattering, and nonshattering states and/or wild, *japonica*, and *indica* rice based on spikelet bases are divergent among the criteria provided by different researchers (Zheng et al., 2007; Pan, 2008; Fuller et al., 2009; Fuller et al., 2010; Pan, 2011; Gross and Zhao, 2014; Zheng et al., 2016), and their diagnostic power for domestication has the potential to be more reliable. More importantly, these macrobotanical remains do not preserve well in early sediments with acid soil; therefore, very few have been recovered from sites dated earlier than 9,000 BP (Zhao, 2011; Qin, 2012; Zhao and Jiang, 2016).

Phytoliths have played an important role in the identification of rice remains recovered from early archaeological sites, due to their high resistance to decomposition (Piperno, 2006; Ball et al., 2016). Double-peaked phytoliths from husks and bulliform phytoliths from rice leaves are both certainly diagnostic indicators of *Oryza* and show variation within and between species. A number of identification criteria based on these phytoliths have been suggested and widely used in the last 20 years (Zhao et al., 1998; Lu et al., 2002; Gu et al., 2013; Wu et al., 2014; Huan et al., 2015; Hilbert et al., 2017; Zuo et al., 2017; Wang et al., 2018b). Although the utility of these methods for distinguishing domesticated from wild rice is still under debate (Fuller et al., 2010; Fuller, 2018), they are recognized as key alternative methodologies besides using morphological domestication data of rice macroremains. Three-dimensional measurements and discriminant function analysis of double-peaked phytoliths are useful in determining the wild/domesticated nature of rice remains; however, double-peaked phytoliths usually present their side and top view under the microscope, which does not meet the requirements of morphometric analysis (present in front view) (Zhao et al., 1998; Gu, 2009), making the work arduous in most cases.

Rice bulliform phytoliths are abundant in rice leaves and are often well preserved and represented in archaeological sediments (Fujiwara, 1993; Wang and Lu, 1993). Generally, bulliform phytoliths in *Oryza* have a distinctive fan shape with numerous scale-like decorations on the half round side (lateral side) (Lu et al., 2002; Wang and Lu, 2012; Gu et al., 2013). Morphological measurements and number of scale-like decorations along the scalloped edge have been employed to distinguish wild *Oryza* species from domesticated ones. Studies on modern rice plants and paddy surface soils have suggested that bulliform phytoliths with ≥9 scale-like decorations were likely domesticated, while those with <9 were generally wild (Lu et al., 2002; Huan et al., 2015). Whether this feature is a useful domestication indicator remains inconclusive and requires further validation; in addition, genetic explanatory mechanisms of bulliform scale-like decoration variation between species remain unclear and deserve further study.

Bulliform shape of rice appears to be under the control of 16 genes (QTLs) (Zheng et al., 2003a) and phytoliths may shift toward larger sizes as a result of genetic mutation during the domestication process (Zheng et al., 2003b; Piperno, 2006; Luo et al., 2016). Some researchers have, therefore, assumed that the bulliforms of domesticated rice are usually larger than wild ones and that the trend of increasing size in rice bulliform phytoliths could reflect domestication of rice (Zheng et al., 2003b, Zheng et al., 2004; Fuller et al., 2007). In recent years, vertical and horizontal lengths (i.e., sizes) of rice bulliforms have increasingly been used as a proxy for tracking the domestication process or determining the degree of domestication at different sites, such as the Tanghu (Zhang et al., 2012), Zhuzhai (Wang et al., 2018b), Shunshanji (Luo et al., 2016), Shangshan, Hehuanshan, and Huxi sites (Zuo et al., 2017; Qiu et al., 2019). However, to date, morphometric data from modern rice plants supporting this method and their assumptions are missing (Pearsall et al., 1995; Zhang and Wang, 1998; Ma and Fang, 2007; Gu et al., 2013), and thus additional evidence is required to test its veracity.

Moreover, some studies have argued that changes in phytolith size may not only be triggered by domestication but also influenced by environmental factors, such as CO_2_ concentrations (Ge et al., 2010), evapotranspiration rates (Issaharou-Matchi et al., 2016), and water levels in the growing habitat (Fuller, 2018). Fuller (2018) indicated that the 16 genes suggested by Zheng et al. (2003a) only explained between 37 and 54% of bulliform variation, suggesting that the environment or growing conditions also play an essential role. These authors further speculated that if shifting bulliform morphology was merely a phenotypic response to environmental conditions, it would be a less useful indicator of domestication. Thus, without data supporting the exclusion of environmental factors, bulliform phytolith measurements alone may not be an accurate identification tool for distinguishing between domesticated and wild *Oryza* species.

In the present study, we tested whether the size of bulliform phytoliths is an effective statistical indicator for distinguishing between wild rice and domesticated rice, based on the comparative analysis of morphometric data from different rice species. In addition, we attempted to examine how growing conditions, especially climate and water levels, influence bulliform size. Finally, we discussed how rice bulliform phytolith morphometry can be used in archaeological research.

## Materials and Methods

**Figure 1:**
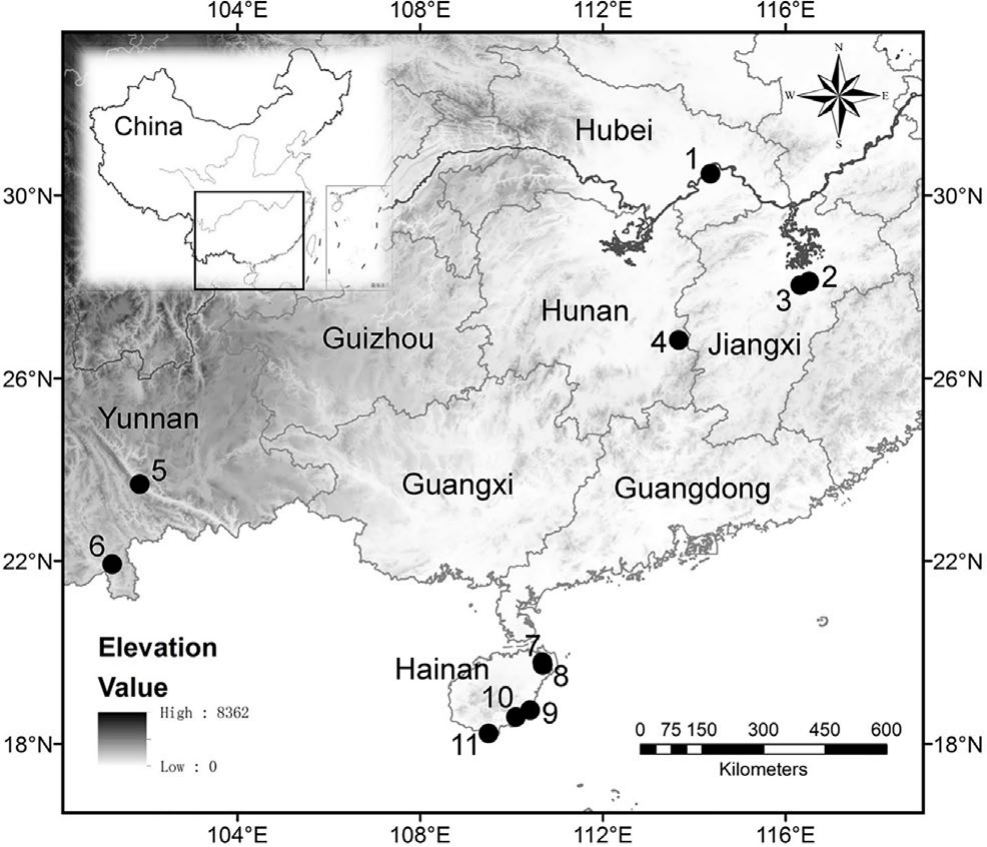
Locations of sample collection sites. Test paddy field in Huazhong Agricultural University, Wuhan, Hubei Province **(1)**; Shuitaoshuxia, Dongxiang, Jiangxi Province **(2)**; Anjiashan, Dongxiang, Jiangxi Province **(3)**; Huli Marsh, Chaling, Hunan Province **(4)**; Yuanjiang protection area of wild rice, Yunnan Province **(5)**; Xishuangbanna Tropical Botanical Garden, CAS, Yunnan Province **(6)**; Hulu village, Wenchang, Hainan Province **(7)**; Tanshen village, Wenchang, Hainan Province **(8)**; Mingxing village, Wanning, Hainan Province **(9)**; Zhangxian village, Lingshui, Hainan Province **(10)**; Luhuitou Park, Sanya, Hainan Province **(11)**.

In the present study, a total of 24 specimens of *Oryza* were sampled. The samples consisted of six specimens of the domestic *O. sativa*, and for the wild species, 16 specimens of *O. rufipogon*, 1 specimen of *O. officinalis*, and 1 specimen of *O. meyeriana* all collected in southern China (**Table 1**; **Figure 1**). All 6 specimens of *O. sativa* and 8 of the 16 specimens of *O. rufipogon* were sampled from the test paddy field belonging to Wuhan Botanical Garden, Chinese Academy of Sciences (CAS), at Huazhong Agricultural University, Wuhan, Hubei Province. Another eight specimens of *O. rufipogon* were sampled from Hainan, Yunnan, Hunan, and Jiangxi Provinces. The specimens of *O. officinalis* and *O. meyeriana* were sampled from Hainan Province.

**Table 1:** Information on the rice plants studied and measured data of vertical length and horizontal length of bulliform phytoliths from the studied samples.

S. no	Field no.	Species	Breed name	Source area	Sampling Province	Sampling site	Locality information	Count number	VL (μm)		HL (μm)	
									Mean	SD	Mean	SD
1	AA31	*O. sativa*	Qi ai zhan	Guangxi	Hubei	Huazhong Agricultural University	30.47°N, 114.36°E, altitude 19m	102	43.29	6.03	38.31	6.26
2	AA36	*O. sativa*	C xiang 517	Guizhou	Hubei	Huazhong Agricultural University	30.47°N, 114.36°E, altitude 19m	102	40.36	6.23	34.81	6.86
3	AA37	*O. sativa*	Bai zhe hu	Guizhou	Hubei	Huazhong Agricultural University	30.47°N, 114.36°E, altitude 19m	102	44.52	6.22	35.97	6.09
4	AA38	*O. sativa*	Bai zhu jing	Guizhou	Hubei	Huazhong Agricultural University	30.47°N, 114.36°E, altitude 19m	101	37.77	4.74	32.78	4.47
5	AA48	*O. sativa*	Wu mang zi ye dao	Heilongjiang	Hubei	Huazhong Agricultural University	30.47°N, 114.36°E, altitude 19m	102	40.13	6.14	32.93	5.06
6	AA49	*O. sativa*	Ji 90 D33	Jilin	Hubei	Huazhong Agricultural University	30.47°N, 114.36°E, altitude 19m	100	42.22	5.59	35.44	5.73
7	Z1	*O. rufipogon*		Dongxiang, Jiangxi	Hubei	Huazhong Agricultural University	30.47°N, 114.36°E, altitude 19m	101	37.89	5.11	32.23	5.62
8	Z12	*O. rufipogon*		Baise, Guangxi	Hubei	Huazhong Agricultural University	30.47°N, 114.36°E, altitude 19m	102	37.06	5.52	30.64	5.16
9	Z13	*O. rufipogon*		Gaozhou, Guangdong	Hubei	Huazhong Agricultural University	30.47°N, 114.36°E, altitude 19m	101	29.89	4.01	24.19	3.99
10	Z14	*O. rufipogon*		Hezhou, Guangxi	Hubei	Huazhong Agricultural University	30.47°N, 114.36°E, altitude 19m	105	36.81	5.34	33.6	5.54
11	Z15	*O. rufipogon*		Zhanjiang, Guangdong	Hubei	Huazhong Agricultural University	30.47°N, 114.36°E, altitude 19m	115	35.39	4.6	32.1	4.48
12	Z23	*O. rufipogon*		Wenchang, Hainan	Hubei	Huazhong Agricultural University	30.47°N, 114.36°E, altitude 19m	110	41.27	7.22	34.08	7.34
13	Z27	*O. rufipogon*		Beihai, Guangxi	Hubei	Huazhong Agricultural University	30.47°N, 114.36°E, altitude 19m	101	38.79	5.82	32.48	5.58
14	Z41	*O. rufipogon*	JW1	India	Hubei	Huazhong Agricultural University	30.47°N, 114.36°E, altitude 19m	110	33.31	4.82	29.04	5.05
15	HL-3	*O. rufipogon*		Wenchang, Hainan	Hainan	Hulu village, Wenchang	19.79°N, 110.68°E, altitude 34m	102	46.53	7.11	40.1	6.32
16	WN-7	*O. rufipogon*		Wanning, Hainan	Hainan	Mingxing village, Wanning	18.74°N, 110.41°E, altitude 10m	100	52.23	6.84	43.05	6.74
17	TS-1	*O. rufipogon*		Wenchang, Hainan	Hainan	Tanshen village, Wenchang	19.73°N, 110.69°E, altitude 21m	100	47.98	7.1	41.58	6.46
18	YNY-1	*O. rufipogon*		Yuanjiang, Yunnan	Yunnan	Yuanjiang protection area of wild rice	23.68°N, 101.86°E, altitude 800m	102	41.63	6.21	34.3	5.04
19	P4	*O. rufipogon*		Chaling, Hunan	Hunan	Huli wetland, Chaling	26.83°N, 113.67°E, altitude 150m	100	41.06	7.32	35.95	6.53
20	DXS-1	*O. rufipogon*		Dongxiang, Jiangxi	Jiangxi	Shuitaoshuxia, Dongxiang	28.11°N, 116.52°E, altitude 47m	100	43.16	5.43	39.62	5.92
21	DXA-1	*O. rufipogon*		Dongxiang, Jiangxi	Jiangxi	Anjiashan, Dongxiang	28.03°N, 116.33°E, altitude 37m	100	41.41	5.21	37.89	5.81
22	XSBN	*O. rufipogon*		Xishuangbanna, Yunnan	Yunnan	Xishuangbanna Tropical Botanical Garden, CAS	21.93°N, 101.26°E, altitude 544m	100	38.34	5.86	33.14	5.22
23	LHT-1	*O. meyeriana*		Sanya, Hainan	Hainan	Luhuitou Park, Sanya	18.23°N, 109.50°E, altitude 132m	34	38.02	5.35	29.37	6.29
24	ZX-345	*O. officinalis*		Lingshui, Hainan	Hainan	Zhangxian village, Lingshui	18.59°N, 110.10°E, altitude 194m	100	51.13	8.77	40.43	7.34

Field collection of rice plants was assisted by a team of investigators from Wuhan Botanical Garden and Nanjing Institute of Geology and Palaeontology, CAS, with the permission of the owner or regulatory body for each location. The criteria for categorizing and naming the species collected were accepted from the classification scheme of the genus *Oryza* in the Flora of China (Liu and Phillips, 2006) (available at http://flora.huh.harvard.edu/china/PDF/PDF22/Oryza.pdf). The specimens of *O. sativa* and *O. rufipogon* in Wuhan were both mature when we collected them between September 21 and 23, 2011, but the ripening rate of *O. rufipogon* was very low due to low temperatures. In Hainan Province, the specimens of *O. rufipogon* were in anthesis and immature while the specimens of *O. officinalis* and *O. meyeriana* were mature and had begun shattering when we collected them between December 1 and 7, 2012. The specimens of *O. rufipogon* in Chaling, Hunan Province, were immature when we collected them between September 18 and 28, 2010. The specimens of *O. rufipogon* in Jiangxi and Yunnan Province were already mature when we collected them during October and November 2010, respectively. All plant samples were preserved at the Institute of Geology and Geophysics, CAS, Beijing.

**Figure 2:**
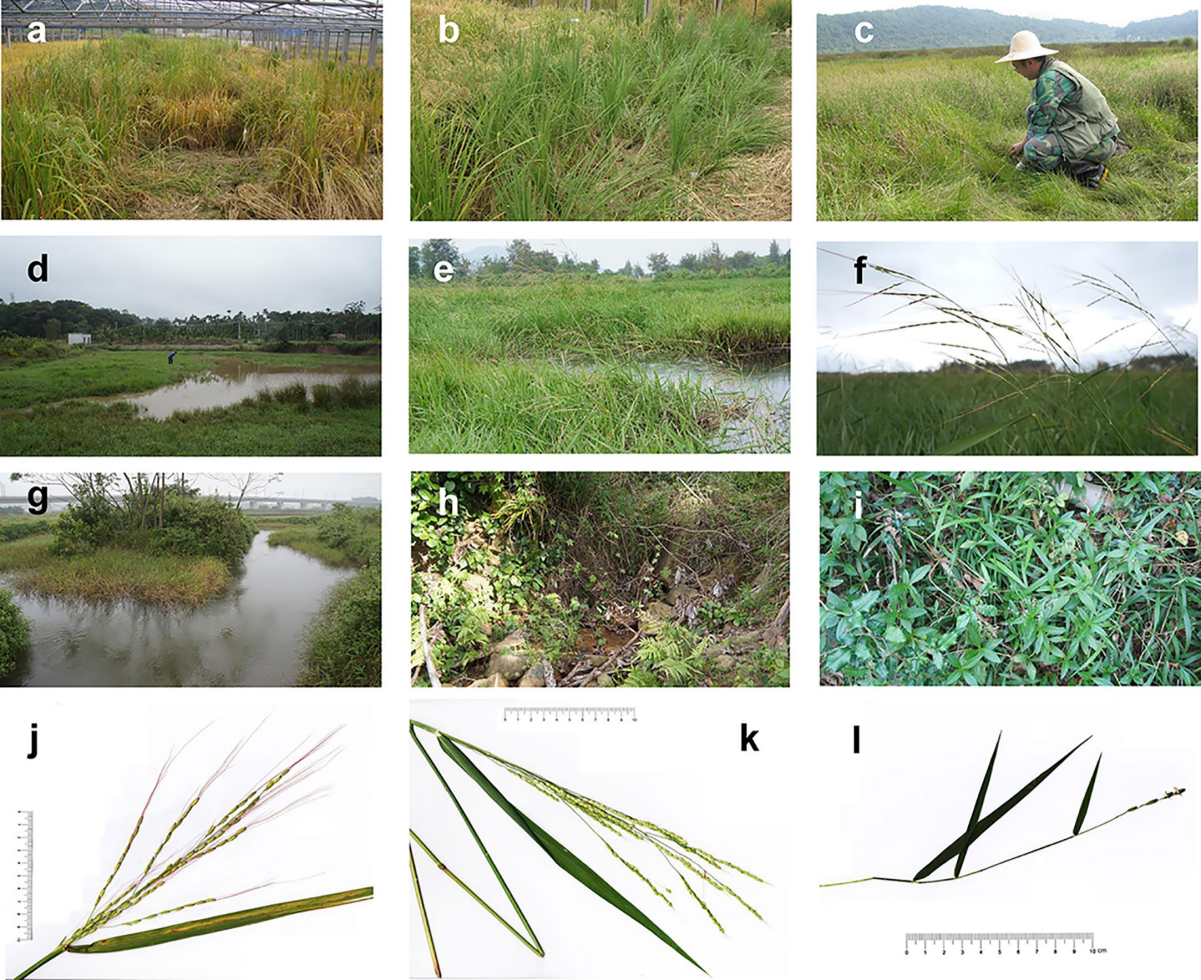
Photos of parts of sampling locations and rice plants. Domesticated rice paddy in Huazhong Agricultural University, Wuhan, Hubei Province (site 1 in **Figure 1**) **(a)**; wild rice paddy in Huazhong Agricultural University, Wuhan, Hubei Province (site 1 in **Figure 1**) **(b)**; site of *Oryza rufipogon* population in Huli Marsh, Chaling, Hunan Province (site 4 in **Figure 1**) **(c)**; site of *O. rufipogon* population in Hulu village, Wenchang, Hainan Province (site 7 in **Figure 1**) **(d)**; site and plants of *O. rufipogon* population in Mingxing village, Wanning, Hainan Province (site 9 in **Figure 1**) **(e, f)**; site of *O. rufipogon* population in Tanshen village, Wenchang, Hainan Province (site 8 in **Figure 1**) **(g)**; site of *O. officinalis* population in Zhangxian village, Lingshui, Hainan Province (site 10 in **Figure 1**) **(h)**; site of *O. meyeriana* population in Luhuitou Park, Sanya, Hainan Province (site 11 in **Figure 1**) **(i)**; plant of *O. rufipogon* in Hainan Province **(j)**; plant of *O. officinalis* in Hainan Province **(k)**; plant of *O. meyeriana* in Hainan Province **(l)**; the pictures of rice plants were taken by Dr. Limi Mao. The individual in **Figure 2c** was Dr. Jianping Zhang who had approved the publication of this image.

There are different hydrological environments among our sampling sites. In Wuhan, specimens of *O. sativa* and *O. rufipogon* were simultaneously cultivated at paddy fields with shallow water (**Figures 2a, b**), which sometimes needs draining to achieve moderate draught during the pustulation and fruiting stage; *O. rufipogon* from Xishuangbanna Tropical Botanical Garden, CAS, Jinghong County, had a similar habitat. In Chaling, *O. rufipogon* grows in the Huli Marsh where there is perennial stagnant water (**Figure 2c**); *O. rufipogon* from Yuanjiang has a similar habitat. In Anjiashan and Shuitaoshuxia, Dongxiang County, *O. rufipogon* grows in a seasonal wetland with 0–100-cm water depth. In Hainan Province, specimens of *O. rufipogon* from Wenchang and Wanning Cities grow in permanent wetlands where there are ponds filled with deep water with 30–150-cm depth (**Figures 2d–g, j**); *O. officinalis* in Lingshui County grows in a ravine stream in the valley and prefers shady and wet habitats (**Figures 2h, k**); *O. meyeriana* in Sanya City grows in an understory bush on a hill and prefers shady and dry habitats (**Figures 2i, l**).

For each rice specimen, we selected all leaf blades from the bottom to the top of a single plant, making sure to keep the leaf blade intact for phytolith extraction. This is because there is significant difference in bulliform phytolith size among different leaf blades of the same plant and different parts of the same leaf blade, with the smaller bulliform phytoliths from the lower leaves (Fuller and Qin, 2009). Bulliform size tends to decrease from leaf base to leaf apex of the same leaf blade (Wang et al., 1997). Therefore, variation in bulliform phytoliths from a few randomly selected rice leaves does not reflect the overall data, and only the selection of all intact leaves can guarantee the representativeness and reliability of the data.

Leaf blades were cleaned with distilled water in an ultrasonic cleaner, and then prepared for wet oxidation: 1) all samples were cut into 1–3-cm pieces and placed in 20 ml of 65% saturated nitric acid for over 12 h, then heated in a water bath for 20 min to oxidize organic materials completely. 2) The solutions were centrifuged at 3,000 rpm for 6 min, decanted and rinsed three times with distilled water, and then rinsed with 95% ethanol until the supernatants were clear. 3) The extracted phytoliths were mounted onto microscopic slides in neutral resin, and the residual samples were transferred to storage vials.

A Leica DM750 light microscope at 600× magnification was used for photomicrography and phytolith counting. One hundred or more bulliform phytoliths, including asymmetric types, were counted in each sample, except for the sample of *O. meyeriana* which produced only a few bulliform phytoliths. Two morphometric parameters, vertical and horizonal lengths (VL and HL), were measured to describe the size of bulliform phytoliths. The measurements were taken from images using the ImageJ software (version 1.48r.). Descriptive statistics of morphometric data were performed using Excel software, and the mean ± SD of each sample was plotted using the Grapher software to perform a comparative analysis. Discriminant function analysis in SPSS 24.0 software was then used to statistically determine the differences in bulliform sizes in different species.

In order to understand whether bulliform phytolith size correlated with environmental conditions and to what degree, a Pearson correlation analysis was performed. Eight environmental variables were chosen: altitude, mean annual precipitation (MAP), July precipitation (MP7), January precipitation (MP1), mean annual temperature (MAT), July temperature (MT7), January temperature (MT1), and relative humidity (HHH). Modern climatic data for the 11 sampling sites (**Figure 1**) were obtained from the nearest meteorological station to each site, since the spatial variation of climatic parameters exhibits a clear gradient across these locations. These data can be collected from the databases (1981–2010) of the National Meteorological Information Center, China (http://data.cma.cn/). Origin 8.5.1 software was used to conduct the correlation analysis of bulliform morphometrics and environmental variables, which were plotted into scatter plots. A linear regression was inserted into these scatter plots, and then the Pearson correlation coefficients (*r*) and significances (*P*) were taken to statistically evaluate the correlation.

## Results

### Morphological Contrast of Bulliform Phytoliths in the Four *Oryza* Species

**Figure 3:**
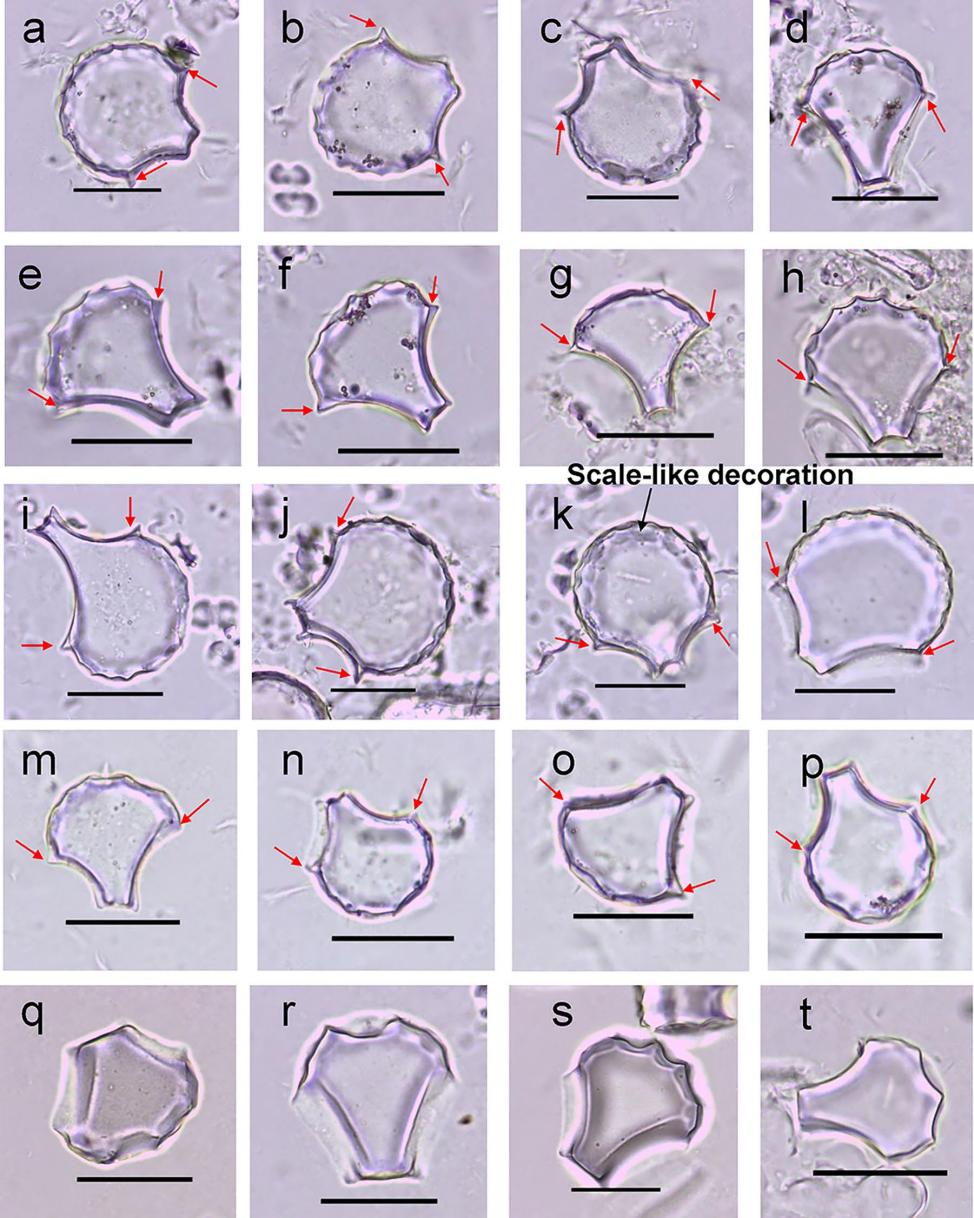
Bulliform phytoliths in some studied *Oryza sativa* and *O. rufipogon* species. Bulliform phytoliths from *O. sativa* (AA31) **(a–d)**; bulliform phytoliths from *O. sativa* (AA48) **(e–h)**; bulliform phytoliths from *O. rufipogon* (HL-3) **(i–l)**; bulliform phytoliths from *O. rufipogon* (Z13) **(m–p)**; bulliform phytoliths from *O. rufipogon* (XSBN) **(q–t)**; scale bar = 30 μm. The red arrows point to the ridge-like tubercle.

Overall, the bulliform phytoliths in *O. sativa* and *O. rufipogon* have similar shapes with an intact circular part of the fan, round arc of the scalloped edge, and ridge-like tubercle on the lateral side (**Figure 3**). Significant intraspecific morphological variation, however, was also found in both *O. sativa* (**Figures 3a–h**) and *O. rufipogon* (**Figures 3i–p**). Moreover, we noted that the bulliform phytoliths from the Xishuangbanna *O. rufipogon* had a very specific shape with a small circular part of the fan, angular scalloped edge, large deep decorations, and without the ridge-like tubercle on the lateral side (**Figures 3q–t**). This shape was not only different from that of *O. sativa* but also distinct among other *O. rufipogon* specimens.

Bulliform phytoliths of *O. officinalis* are mostly large and full in shape with a rounded arc of the scalloped edge, irregular large deep decorations, longer handles, and a shorter intact circular part of the fan but without the ridge-like tubercle on the lateral side (**Figure S1**). From an overall perspective, this shape is similar to that of *O. sativa* and *O. rufipogon*.

Bulliform phytoliths of *O. meyeriana* are generally long and thin and not full in shape like the other varieties. These phytoliths are very small and appear similar to a teardrop or nail, with an angular scalloped edge, small irregular, but deep decorations, a longer handle, and shorter circular part of the fan not intact, without the ridge-like tubercle on the lateral side (**Figure S2**). This shape is different from that of the other three *Oryza* species.

### Morphometric Analysis of Bulliform Phytoliths in the Four *Oryza* Species

Overall, our morphometric data demonstrated that the bulliforms of domesticated rice were not always larger than wild ones, and there was no significant difference in size. **Table 1** shows the mean values of vertical length (VL) and horizontal length (HL) of bulliform phytoliths from the studied samples. For original measured data, see the **Datasheet S1**.

In the six *O. sativa* specimens, the maximum mean of VL of bulliform phytoliths was 44.52 ± 6.22 μm (AA37), while the minimum was 37.77 ± 4.74 μm (AA38); the maximum mean of HL of bulliform phytoliths was 38.31 ± 6.26 μm (AA31), while the minimum was 32.78 ± 4.47 μm (AA38). In all 609 bulliform phytoliths from *O. sativa*, the maximum VL was 60.27 μm occurring in sample AA36, while the minimum was 25.25 μm occurring in sample AA38; the maximum HL was 61.96 μm occurring in sample AA31, while the minimum was 20.94 μm occurring in sample AA48.

In the 16 *O. rufipogon* specimens, the maximum mean of VL of bulliform phytoliths was 52.23 ± 6.84 μm (WN-7), while the minimum was 29.89 ± 4.01 μm (Z13); the maximum mean of HL of bulliform phytoliths was 43.05 ± 6.74 μm (WN-7), while the minimum was 24.19 ± 3.99 μm (Z13). In all 1,649 bulliform phytoliths, the maximum VL was 70.11 μm occurring in sample WN-7, while the minimum was 21.30 μm occurring in sample Z13; the maximum HL was 59.81 μm occurring in sample WN-7, while the minimum was 15.54 μm occurring in sample Z13.

In the only specimen of *O. officinalis*, the mean values of VL and HL of bulliform phytoliths were 51.13 ± 8.77 and 40.43 ± 7.34 μm, respectively. In all 100 bulliform phytoliths, the maximum VL was 80.00 μm, while the minimum was 32.94 μm; the maximum HL was 62.76 μm, while the minimum was 25.17 μm.

In the only specimen of *O. meyeriana*, the mean values of VL and HL of bulliform phytoliths were 38.02 ± 5.35 and 29.37 ± 6.29 μm, respectively. In all 34 bulliform phytoliths, the maximum VL was 50.59 μm, while the minimum was 30.49 μm; the maximum HL was 44.26 μm, while the minimum was 18.18 μm.

**Figure 4:**
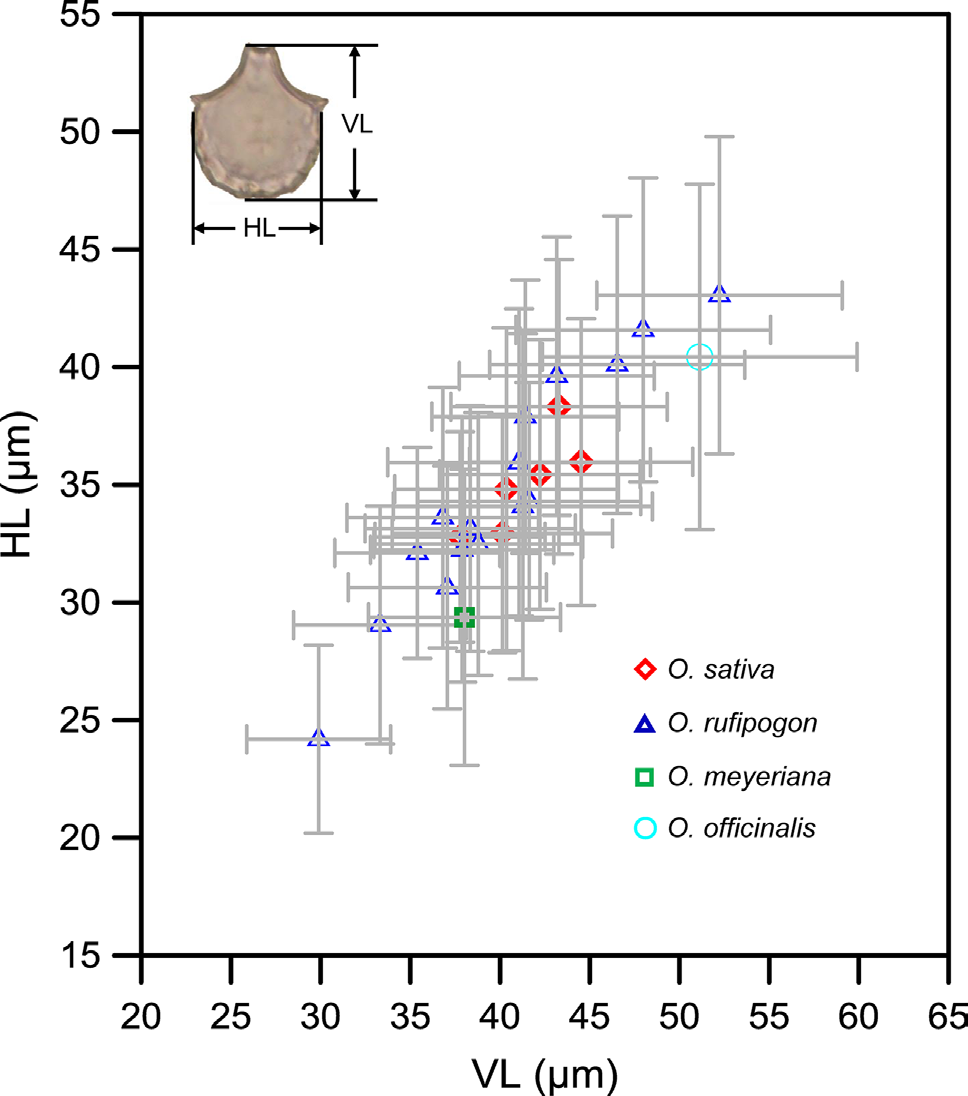
*Oryza* bulliform phytolith measurements from the studied species. VL, vertical length; HL, horizontal length of rice bulliform; gray error bar represents ± 1 SD.

**Figure 4** presents a comparison of the sizes of bulliform phytoliths from the studied rice species. The results revealed that the values of VL and HL from *O. rufipogon* were scattered and widely overlapped with the other three *Oryza* species. Although the bulliform sizes of *O. sativa* were larger compared with *O. meyeriana*, they partly overlapped with *O. rufipogon* (mean VL: 37–45 μm; HL: 32–40 μm) and were significantly smaller than those of *O. officinalis*. The bulliform size of *O. meyeriana* was smaller compared with other *Oryza* species and only larger than a few specimens of *O. rufipogon*. The bulliform size of the specimen of *O. officinalis* was larger, exceeding most of the studied samples.

**Figure 5:**
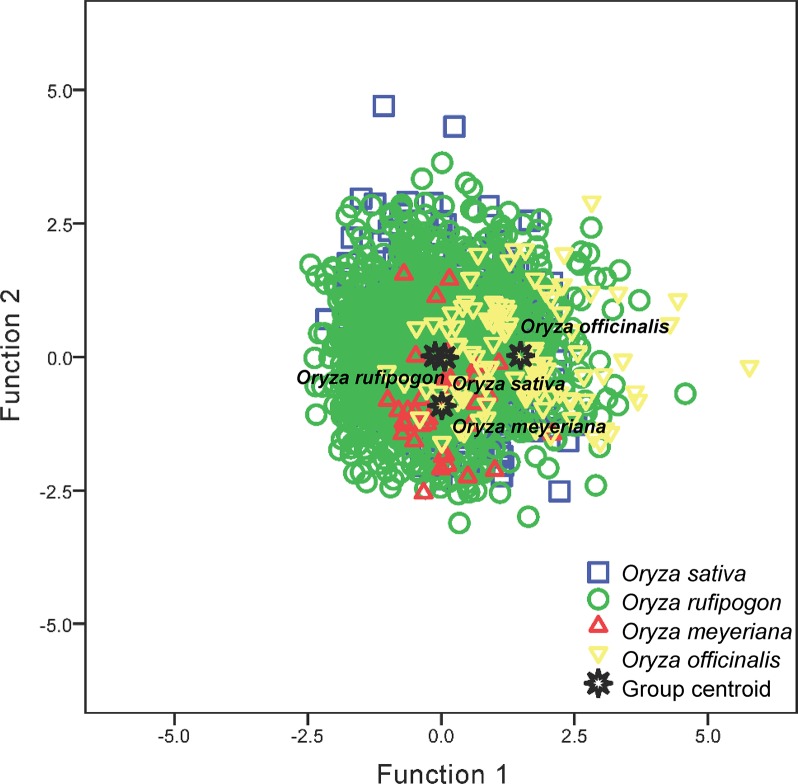
Discriminant function analyses of *Oryza sativa*, *O. rufipogon*, *O. meyeriana*, and *O. officinalis*.

Parameters VL and HL were important and used in the discriminant function analysis (**Table S1**). The following two canonical discriminate functions were used in the analysis: function 1 explained 89.5% of the variance, and function 2 explained 10.5% of the variance. Parameter VL had the largest absolute correlation with function 1, indicating that it contributed most to function 1; parameters HL had the largest absolute correlation with function 2, indicating that it contributed more to function 2 (**Table S1**). These two functions were used to plot the data (**Figure 5**). Four groups without distinct centroids were obtained; *O. sativa* had a clear intersection with *O. rufipogon*, whereas there was a slight distinction for *O. officinalis* and *O. meyeriana* with regard to the other species. The accuracy of the classification was ascertained by cross validating the results (**Table 2**). Only 36.8% of the original data and 36.7% of the cross-validated data were correctly classified, suggesting that the discriminant functions obtained using parameters VL and HL could not be successfully used to discriminate *O. sativa*, *O. rufipogon*, *O. meyeriana*, and *O. officinalis*. Thus, it supported the conclusion that there were no significant differences in bulliform sizes between wild and domesticated rice.

**Table 2:** Classification results of the discriminant function analysis.

			Predicted membership				Total
			*O. sativa*	*O. rufipogon*	*O. meyeriana*	*O. officinalis*	
Original	Count	*O. sativa*	109	232	156	112	609
		*O. rufipogon*	226	678	447	298	1,649
		*O. meyeriana*	4	4	24	2	34
		*O. officinalis*	14	4	13	69	100
	Percent (%)	*O. sativa*	17.9	38.1	25.6	18.4	100
		*O. rufipogon*	13.7	41.1	27.1	18.1	100
		*O. meyeriana*	11.8	11.8	70.6	5.9	100
		*O. officinalis*	14	4	13	69	100
Cross-validated	Count	*O. sativa*	107	233	157	112	609
		*O. rufipogon*	226	678	447	298	1,649
		*O. meyeriana*	4	4	24	2	34
		*O. officinalis*	14	4	13	69	100
	Percent (%)	*O. sativa*	17.6	38.3	25.8	18.4	100
		*O. rufipogon*	13.7	41.1	27.1	18.1	100
		*O. meyeriana*	11.8	11.8	70.6	5.9	100
		*O. officinalis*	14	4	13	69	100

Thirty-six point eight percent of original grouped cases correctly classified. Cross validation is done only for those cases in the analysis. In cross validation, each case is classified by the functions derived from all cases other than that case. Of cross-validated grouped cases, 36.7% are correctly classified.

**Figure 6:**
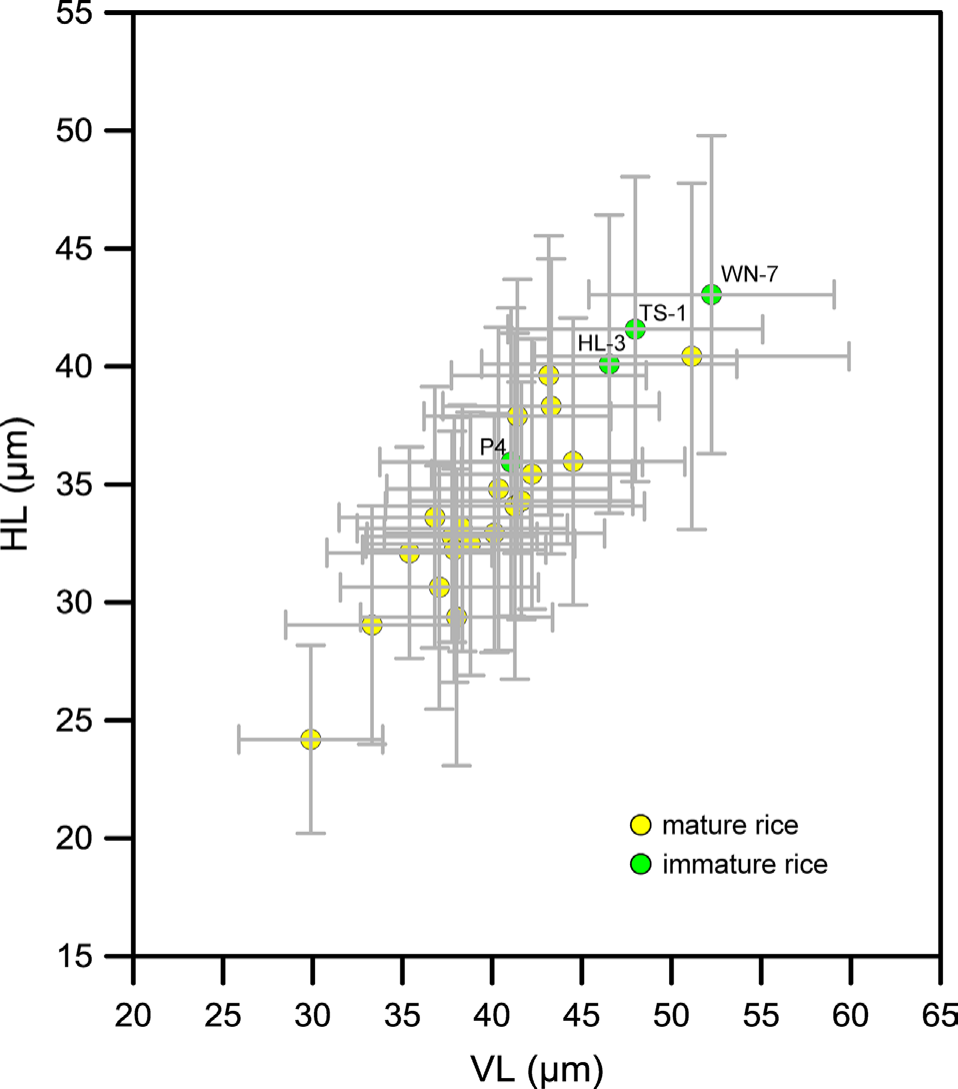
Contrast of *Oryza* bulliform phytolith sizes from the studied specimens depending on whether they were mature or immature. The sample codes in the figure refer to the field numbers: specimens WN-7, TS-1, and HL-3 were *O. rufipogon* from Wanning and Wenchang, Hainan Province, and specimen P4 was *O. rufipogon* from Chaling, Hunan Province.

The 24 specimens of rice were then divided into two groups: mature and immature (**Figure 6**). As can be seen, the sizes of bulliform phytoliths from mature rice were scattered and partly overlapped with the immature rice. In contrast, bulliform phytoliths of immature rice were slightly larger than most mature species. There was no significant difference in bulliform size between mature and immature rice species.

**Figure 7:**
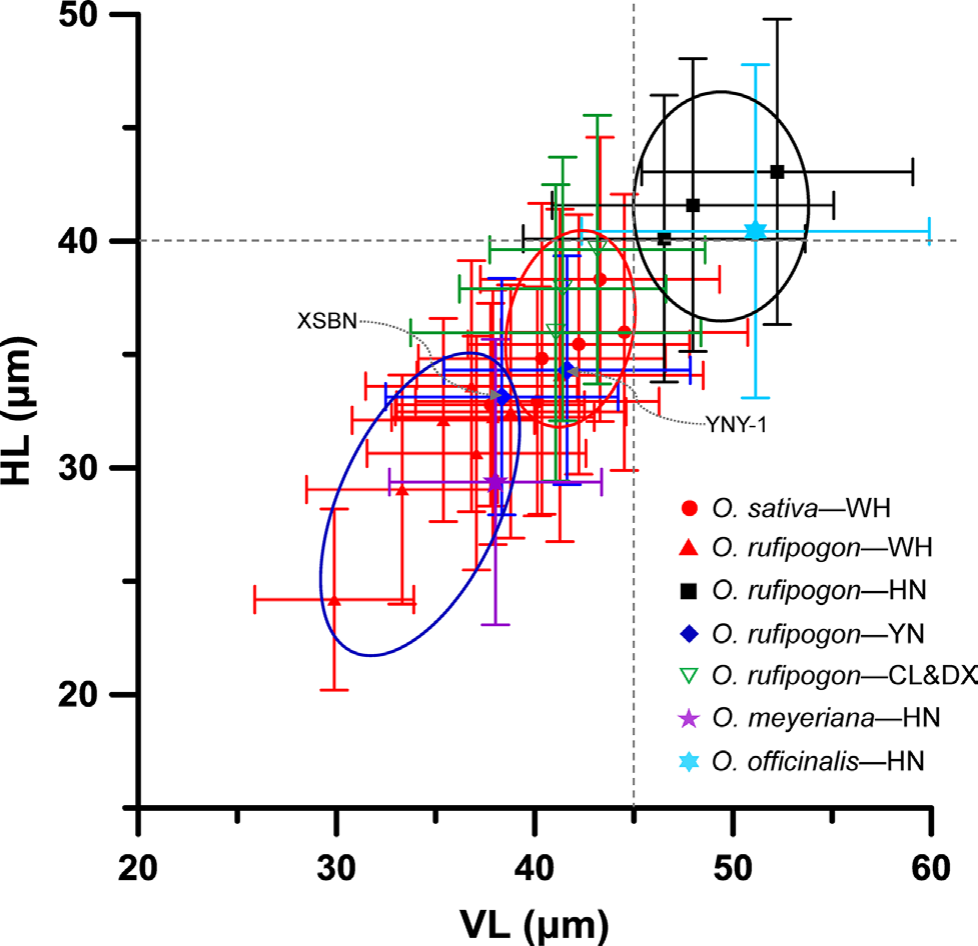
Contrast of *Oryza* bulliform phytolith sizes from the studied specimens in terms of their growing regions. WH, Wuhan; HN, Hainan; YN, Yunnan; CL, Chaling; DX, Dongxiang; XSBN, *O. rufipogon* from Xishuangbanna; YNY-1, *O. rufipogon* from Yuanjiang.

We further compared the bulliform size of rice species in terms of their growing region (**Figure 7**). It was found that the bulliforms of *O. rufipogon* and *O. officinalis* growing in the tropical Hainan were the largest with mean vertical and horizontal lengths greater than 45 and 40 μm, respectively. The bulliforms of *O. rufipogon* growing in Chaling, Dongxiang, and Yuanjiang and *O. sativa* growing in Wuhan were similar in size, with mean VL and HL ranges of 40–45 and 33–40 μm, respectively. *O. rufipogon* growing in Wuhan and Xishuangbanna and *O. meyeriana* growing in Hainan, have the smallest bulliform phytoliths with mean VL and HL ranges of 33–39 and 29–34 μm, respectively. It is noted that *O. rufipogon* and *O. officinalis* in the Hainan population have the most favorable habitat in terms of water availability, with permanent deep water or perennial ravine streams; *O. rufipogon* in Chaling, Dongxiang, and Yuanjiang grow in marshes and seasonal wetlands where stagnant water converges at the roots of rice; *O. rufipogon* in Wuhan and Xishuangbanna grow in paddy fields with shallow water, which is occasionally drained to maintain relatively dry habitats; *O. meyeriana* in Hainan prefers a drier environment.

**Figure 8:**
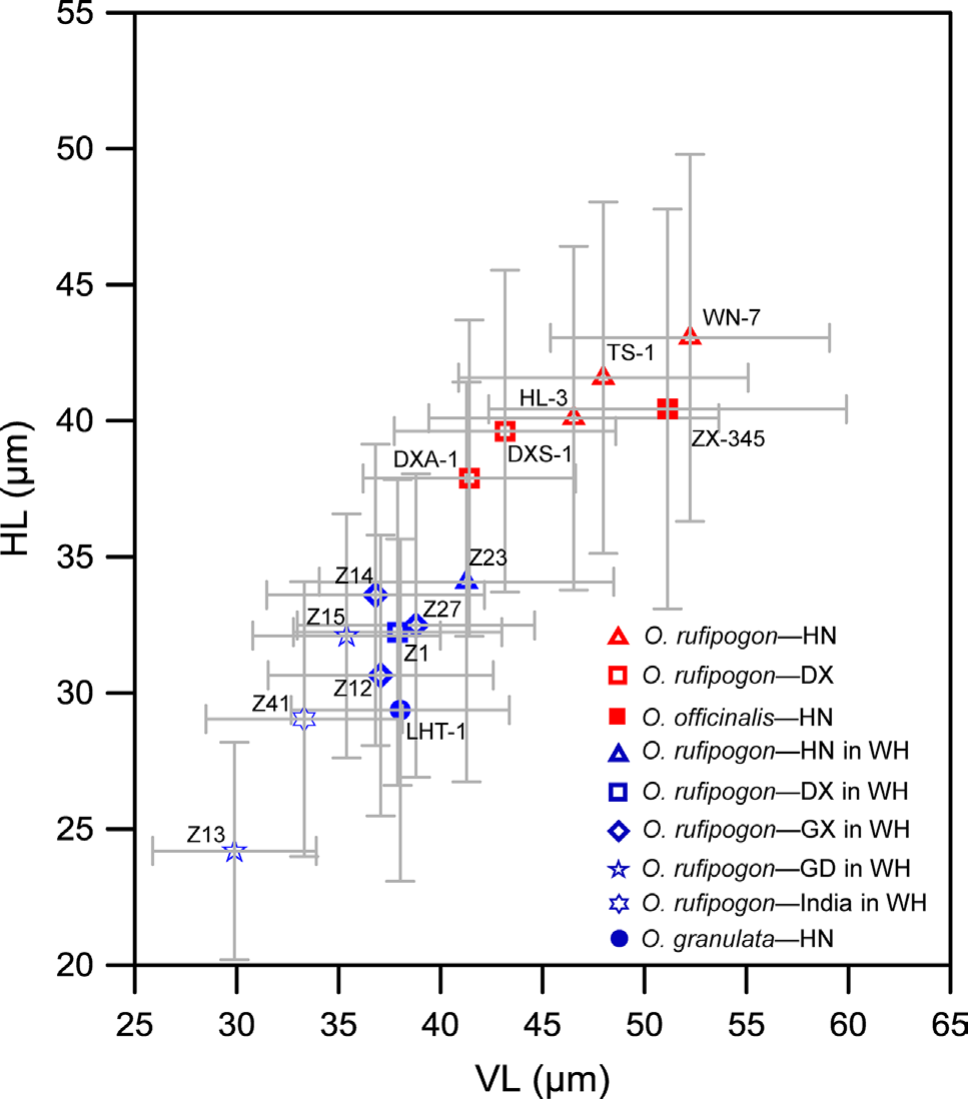
Contrast of bulliform phytolith sizes from some studied wild rice in terms of water levels in their habitats. Red represents wet habitats; blue represents relatively dry habitats. The sample codes in the figure refer to the field numbers. WH, Wuhan; HN, Hainan; DX, Dongxiang; GX, Guangxi; GD, Guangdong.

In addition, the effect of habitat wetness on bulliform phytolith size was investigated. We found that the bulliform phytolith size of specimens of *O. rufipogon* native to the warmer and wetter sites of Wenchang, Hainan (field no. Z23), and Dongxiang, Jiangxi (field no. Z1), and cultivated in the paddy fields in Wuhan, was smaller than that of native species (**Figure 8**). Similarly, in the Wuhan paddy field, the species of *O. rufipogon* native to the warmer and wetter sites of Guangdong (field nos. Z13, Z15), Guangxi (field nos. Z12, Z14, Z27), and India (field no. Z41) also have smaller bulliforms (**Figure 8**). In the tropical Hainan, under parallel climate conditions, *O. officinalis* in the aquatic environment has a significantly larger bulliform size than those of *O. meyeriana* in a dry habitat (**Figure 8**). Therefore, the sizes of bulliform phytoliths from wild rice with preferable water habitats were mostly larger than those of wild rice under relatively dry conditions.

**Figure 9:**
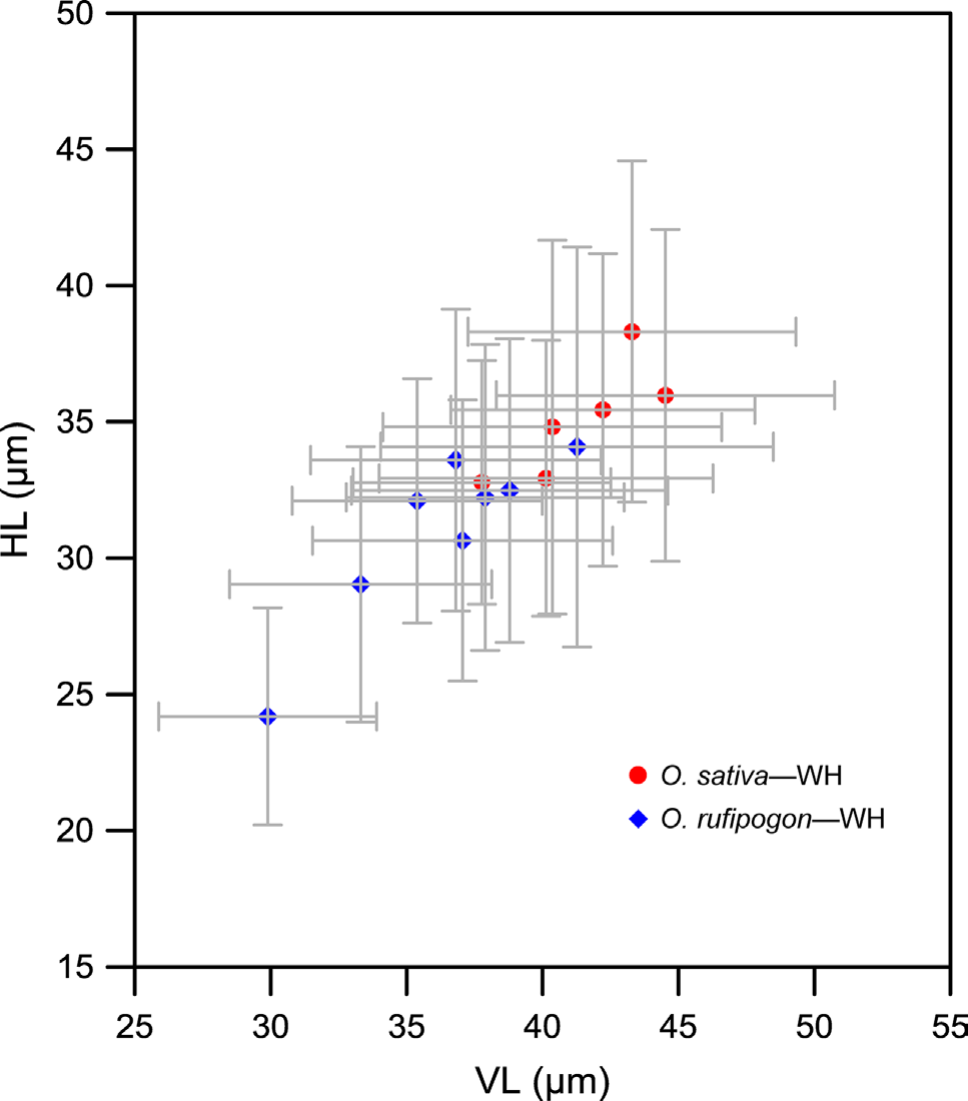
Contrast of bulliform phytolith sizes from domesticated and wild rice growing at the adjacent test paddy field in Wuhan. Demonstrating that the bulliform phytoliths of these domesticated rice specimens were generally larger compared with wild specimens (*Oryza rufipogon*) in the same environment. WH, Wuhan.

Finally, **Figure 9** shows the comparison of bulliform phytolith sizes between *O. sativa* and *O. rufipogon* growing in the adjacent test paddy field in Wuhan. For *O. sativa*, the ranges of mean VL and HL of bulliforms fell into 37–45 and 32–48 μm, respectively. For *O. rufipogon*, the mean VL and HL ranges were 29–41 and 24–34 μm, respectively. Thus, our data also indicated that the bulliform phytoliths of *O. rufipogon* may be generally smaller than those of *O. sativa* if they were artificially grown in the same environment.

### Correlation Analysis of Bulliform Morphometrics and Environmental Variables

**Figure 10:**
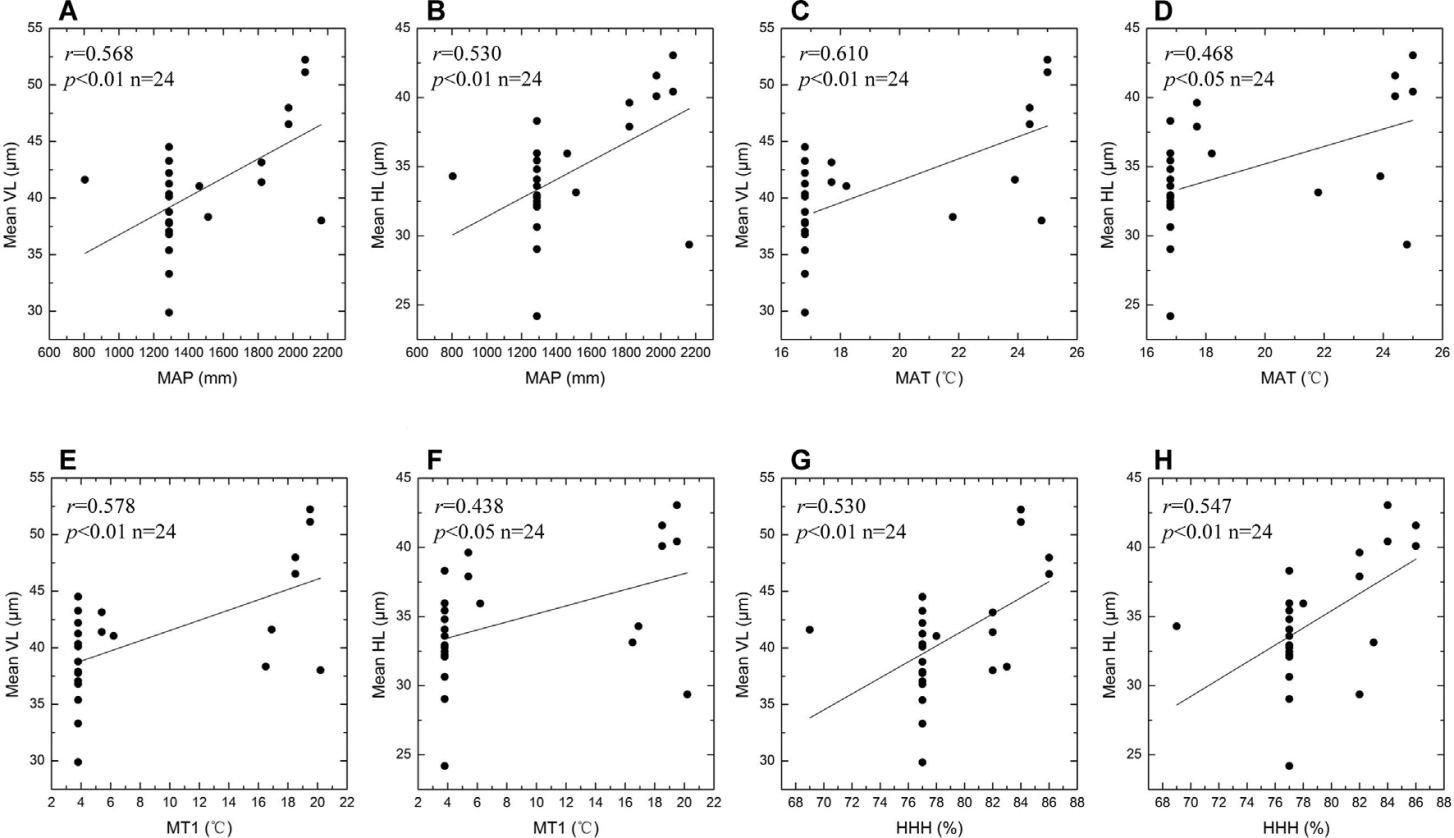
Scatter plots of bulliform phytolith sizes as defined by VL and HL for studied species *versus* different climatic variables. Mean VL values *vs*. observed MAP values **(A)**; mean HL values *vs*. observed MAP values **(B)**; mean VL values *vs*. observed MAT values **(C)**; mean HL values *vs*. observed MAT values **(D)**; mean VL values *vs*. observed MT1 values **(E)**; mean HL values *vs*. observed MT1 values **(F)**; mean VL values *vs*. observed HHH values **(G)**; mean HL values *vs*. observed HHH values **(H)**. Linear regression analysis of these data indicates significant correlation.

**Figure 11:**
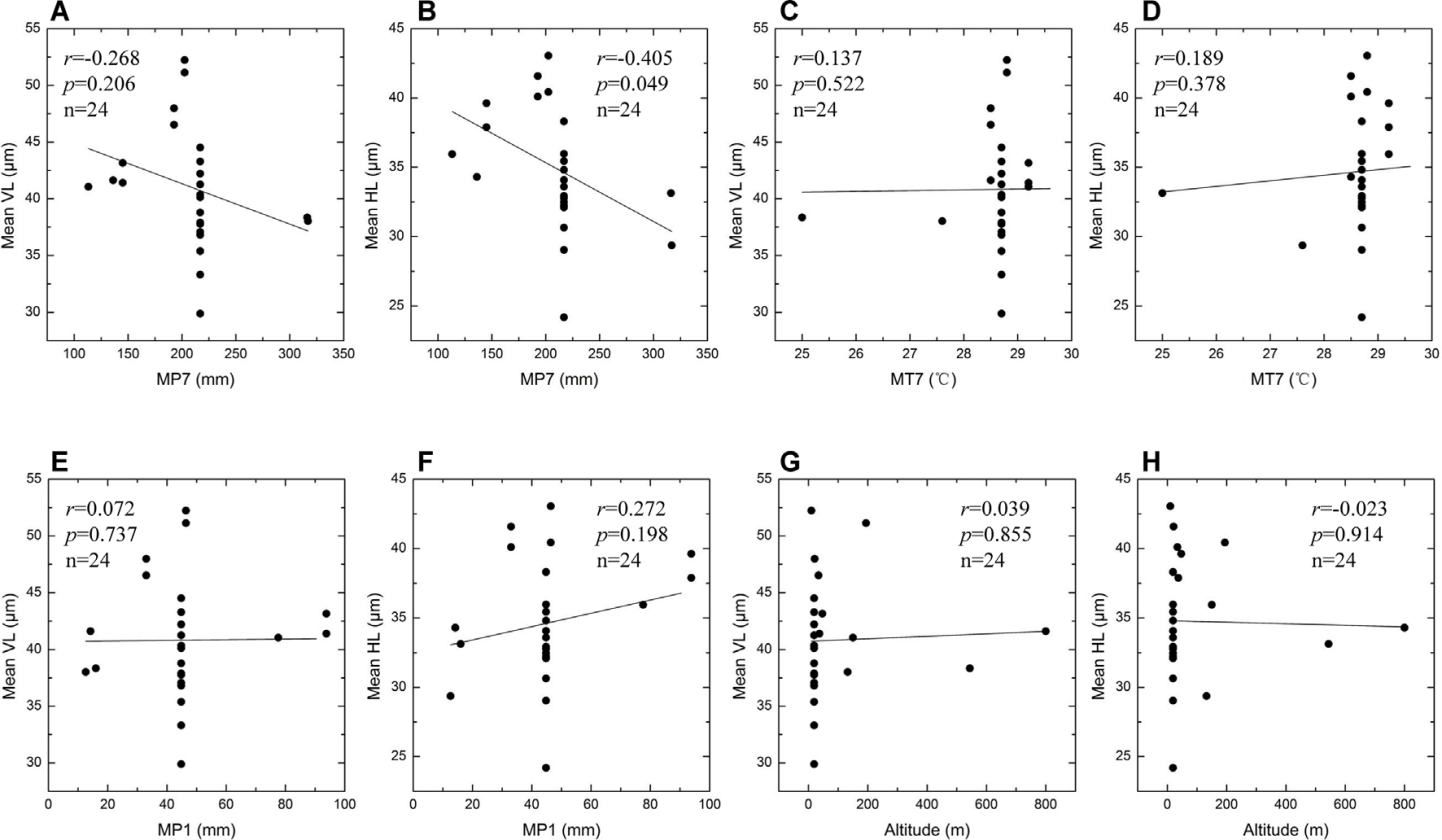
Scatter plots of bulliform phytolith sizes as defined by VL and HL for studied species *versus* different environmental variables. Mean VL values *vs*. observed MP7 values **(A)**; mean HL values *vs*. observed MP7 values **(B)**; mean VL values *vs*. observed MT7 values **(C)**; mean HL values *vs*. observed MT7 values **(D)**; mean VL values *vs*. observed MP1 values **(E)**; mean HL values *vs*. observed MP1 values **(F)**; mean VL values *vs*. altitude values **(G)**; mean HL values *vs*. altitude values **(H)**. Linear regression analysis of these data indicates no significant correlation.

Summary statistics for the eight environmental variables of different sites are given in **Table 3**. The VL and HL values of bulliform phytoliths plotted against the different environmental variables and the results are shown in **Figure 10** and **Figure 11**. MAP, MAT, MT1, and HHH had a positive correlation (*r* = 0.438–0.610) with VL and HL, and the linear regression analysis revealed that these correlations were significant (*p* < 0.01 or 0.05) (**Figure 10**), demonstrating that the bulliform phytolith sizes are affected by changes in these climatic parameters. In contrast, other environmental characteristics, including MP7, MT7, MP1, and altitude, were not significantly correlated with changes in bulliform sizes (**Figure 11**).

**Table 3:** Summary of the environmental variables in the 11 sampling sites used for correlation analysis.

S. no.	Sampling site	Meteorological stations (MS)	Code of MS	MAP (mm)	MP1 (mm)	MP7 (mm)	MAT (°C)	MT1 (°C)	MT7 (°C)	HHH (%)	Altitude (m)
1	Huazhong Agricultural University, Wuhan, Hubei Province	Xinzhou	57492	1,287.8	44.8	216.9	16.8	3.8	28.7	77	19
2	Shuitaoshuxia, Dongxiang, Jiangxi Province	Yujiang	58616	1,819.5	93.8	145	17.7	5.4	29.2	82	47
3	Anjiashan, Dongxiang, Jiangxi Province	Yujiang	58616	1,819.5	93.8	145	17.7	5.4	29.2	82	47
4	Huli Marsh, Chaling, Hunan Province	Chaling	57882	1,461.9	77.6	113.2	18.2	6.2	29.2	78	150
5	Yuanjiang, Yunnan Province	Yuanjiang	56966	804.3	14.2	136.1	23.9	16.9	28.5	69	800
6	Xishuangbannan Tropical Botanical Garden, CAS, Yunnan Province	Mengla	56969	1,513	16	316.2	21.8	16.5	25	83	544
7	Hulu village, Wenchang, Hainan Province	Wenchang	59856	1,975	33	192.6	24.4	18.5	28.5	86	34
8	Tanshen village, Wenchang, Hainan Province	Wenchang	59856	1,975	33	192.6	24.4	18.5	28.5	86	21
9	Mingxing village, Wanning, Hainan Province	Wanning	59951	2,070.3	46.4	202.4	25	19.5	28.8	84	10
10	Zhangxian village, Lingshui, Hainan Province	Wanning	59951	2,070.3	46.4	202.4	25	19.5	28.8	84	194
11	Luhuitou Park, Sanya, Hainan Province	Baoting	59945	2,162.8	12.6	316.9	24.8	20.2	27.6	82	132

MAP, Mean annual precipitation; MP1, January precipitation; MP7, July precipitation; MAT, mean annual temperature; MT1, January temperature; MT7, July temperature; HHH, relative humidity. Data of MAP, MAT, and HHH are obtained from the dataset of annual surface observation values in individual years (1981–2010) in China (http://data.cma.cn/data/cdcdetail/dataCode/A.0029.0005.html); data of MP1, MP7, MT1, and MT7 are obtained from the dataset of monthly surface observation values in individual years (1981–2010) in China (http://data.cma.cn/data/cdcdetail/dataCode/A.0029.0004.html). These datasets belong to the National Meteorological Information Center, China, and are available online.

## Discussion and Conclusions

### Causes of Variations in Rice Bulliform Phytolith Morphometry

The results of the present study indicate that morphometric measurements of bulliform phytoliths from wild and domesticated rice widely overlap (**Figure 4**), exhibiting little diagnostic potential for taxonomic identification at the species level. These results thus support the conclusion that the morphometry of bulliform phytoliths is not as informative for distinguishing between domesticated rice and wild rice, as suggested by previous studies (e.g., Pearsall et al., 1995; Wang and Lu, 2012; Gu et al., 2013). However, the above morphometric data have often been overlooked, and an increasing number of studies have recently used bulliform phytolith size as a proxy to track the rice domestication process (Zhang et al., 2012; Luo et al., 2016; Zuo et al., 2017; Qiu et al., 2019). The bulliform phytoliths from domesticated rice really were larger than those from wild ones in the same test paddy field in Wuhan city (**Figure 9**), possibly indicating that domestication may result in an increase in bulliform phytolith size. The genetic and phylogenetic signal for this bulliform size variation has not been well revealed to date. Furthermore, this increase did not necessarily result from domestication and may be caused by other factors.

Previous studies have suggested that two factors, plant maturity and environmental conditions, may affect rice bulliform phytolith size. It is suggested that the bulliform phytoliths from mature rice leaves are usually larger than those from immature leaves (Zheng et al., 2003a; Qin et al., 2006; Fuller et al., 2007). This hypothesis may be real for the plants growing in the same location, but is not supported when comparing bulliform size of rice species from different sites (**Figure 6**). Because environmental conditions are seemingly much more important, and the effect of degree of maturity could be ignored when multiple environmental factors are considered. According to the results of the present study (**Figures 10** and **11**), from a statistical point of view, we can conclude that the larger rice bulliform phytolith sizes, as defined by the higher VL and HL values, likely occurred at the locations with higher temperature and precipitation. Therefore, the increasing trend in rice bulliform phytolith size in some archaeological records (Zheng et al., 2003b; Zheng et al., 2004; Luo et al., 2016; Zuo et al., 2017; Qiu et al., 2019) may also be caused by climatic changes during the early and middle Holocene when temperature and precipitation were gradually rising.

The present study also revealed that the growing microenvironment, such as water environment, can also influence the size of rice bulliform phytoliths. Rice growing under wetter conditions usually produced larger bulliform phytoliths than those growing under drier conditions (**Figures 7** and **8**). Therefore, except climate regimes, changes in wet/dry habitat for rice should be considered for the use of bulliform size to track the process of rice domestication.

It should be pointed out that the present study just revealed the hydrothermal condition as one of the environmental factors influencing rice bulliform size. Some factors such as plant genotypes, soil fertility, light period length on photosynthesis, and other abiotic factors may also cause these variations, which were not controlled or excluded for this study. Conditional plantation experiment under controlling environments and genotypes in test paddy field is needed to further test if and how water levels and temperature can affect the bulliform phytolith size.

### Implications for Archaeology of Rice Domestication

Changes in bulliform phytolith size of rice are regulated not only by domestication, which possibly represents genetic changes, but also by environmental factors. Given that environmental factors influence bulliform phytolith size of rice and that the role of genetic background has not yet been firmly established similar to established domestication traits such as non-shattering and increased seed size, bulliform measurement was considered as a semi-domestication trait (Fuller and Qin, 2009). Therefore, the use of rice bulliform phytolith size as an index for determining domesticated plants from their wild ancestors should be conditional. In other words, if the increasing size trend of bulliform phytoliths is used to reveal the process of rice domestication, the influence of hydrothermal conditions should be excluded first.

For further archaeological use of this index, we suggest that: 1) if the time series of rice bulliform phytolith size from a region is long, then the climatic changes (fluctuations in temperature and precipitation) through time should be considered, and the results from quantitative reconstructions of paleoclimate could be used as an independent variable to explain bulliform size variation; 2) the spatial scale of studied regions should be small and without a clear climatic gradient; 3) parallel comparison of rice domestication processes in different regions using bulliform size should consider climatic differences between the regions; and 4) the changes in rice arable systems (wet/dry growing conditions) in any studied archaeological sites should be first revealed using the promising sensitive/fixed phytolith morphotype model defined by Weisskopf et al. (2015).

Even though the influence of environmental factors has been controlled or excluded, and rice bulliform phytoliths shifting toward larger sizes are interpreted as reflecting the domestication process, it is still not possible to provide a determinate range of bulliform size for identifying domesticated rice, due to the wide overlap observed in the bulliform morphometric data between modern wild and domesticated rice (**Figure 4**). Thus, rice bulliform phytolith size is a supporting rather than conclusive proxy for determining the domesticated status of rice in archaeological research. Combination of bulliform phytolith size with other established criteria can provide precise identification of wild and domesticated rice.

Finally, notably, frequent gene exchange occurs between domesticated and wild rice, and there is a co-evolutionary relationship between them (Song et al., 2006; Zhao et al., 2010; Ge and Sang, 2011; Choi et al., 2017). Recent large-scale genomic analysis showed that *O. rufipogon* populations are widely affected by the gene flow of domesticated rice, and that the existing *O. rufipogon* are actually a hybrid swarm (Wang et al., 2017). This indicates that it is difficult to rule out the interference of the domesticated rice gene flow when using the existing *O. rufipogon* species as a reference for phytolith morphological analysis. Wild rice populations in the region with higher rice farming intensity are more affected by the introgression of the domesticated rice gene, and the genetic relationship with domesticated rice is closer (Song et al., 2003; Song et al., 2006), leading to the possibility of bias in the morphometric measurements of their bulliform phytoliths. Further research utilizing archaeological rice remains combined with ancient DNA analysis (e.g., Tanaka et al., 2010; Castillo et al., 2016), may reduce the interference of domesticated rice gene flow, and thus generate credible results to establish suitable criteria for distinguishing between domesticated rice and wild rice.

## Data Availability

The datasets generated during the current study are available from the corresponding author on reasonable request.

## Author Contributions

HL and CW designed research. JZ, CW, and LM collected samples. CW and JZ performed experiments. CW and YG analyzed data. CW and HL wrote the paper. All authors read and approved the final manuscript.

## Funding

This work is supported by the National Natural Science Foundation of China (Grant Nos. 41701233, 41830322 and 41430103), the Strategic Priority Research Program of Chinese Academy of Sciences (Grant No. XDB26000000), and the Youth Top Talent Program of the Education Department of Hebei Province (Grant No. BJ2018118).

## Conflict of Interest Statement

The authors declare that the research was conducted in the absence of any commercial or financial relationships that could be construed as a potential conflict of interest.

## Supplementary Material

Datasheet S1 Morphometric data of bulliform phytoliths from the 24 studied samples.
