# Supplementary figures and images for: Bulliform Phytolith Size of Rice and Its Correlation With Hydrothermal Environment: A Preliminary Morphological Study on Species in Southern China
Source: Front Plant Sci. 2019 Aug 22;10:1037. doi: 10.3389/fpls.2019.01037 (PMC6735168; doi:10.3389/fpls.2019.01037)

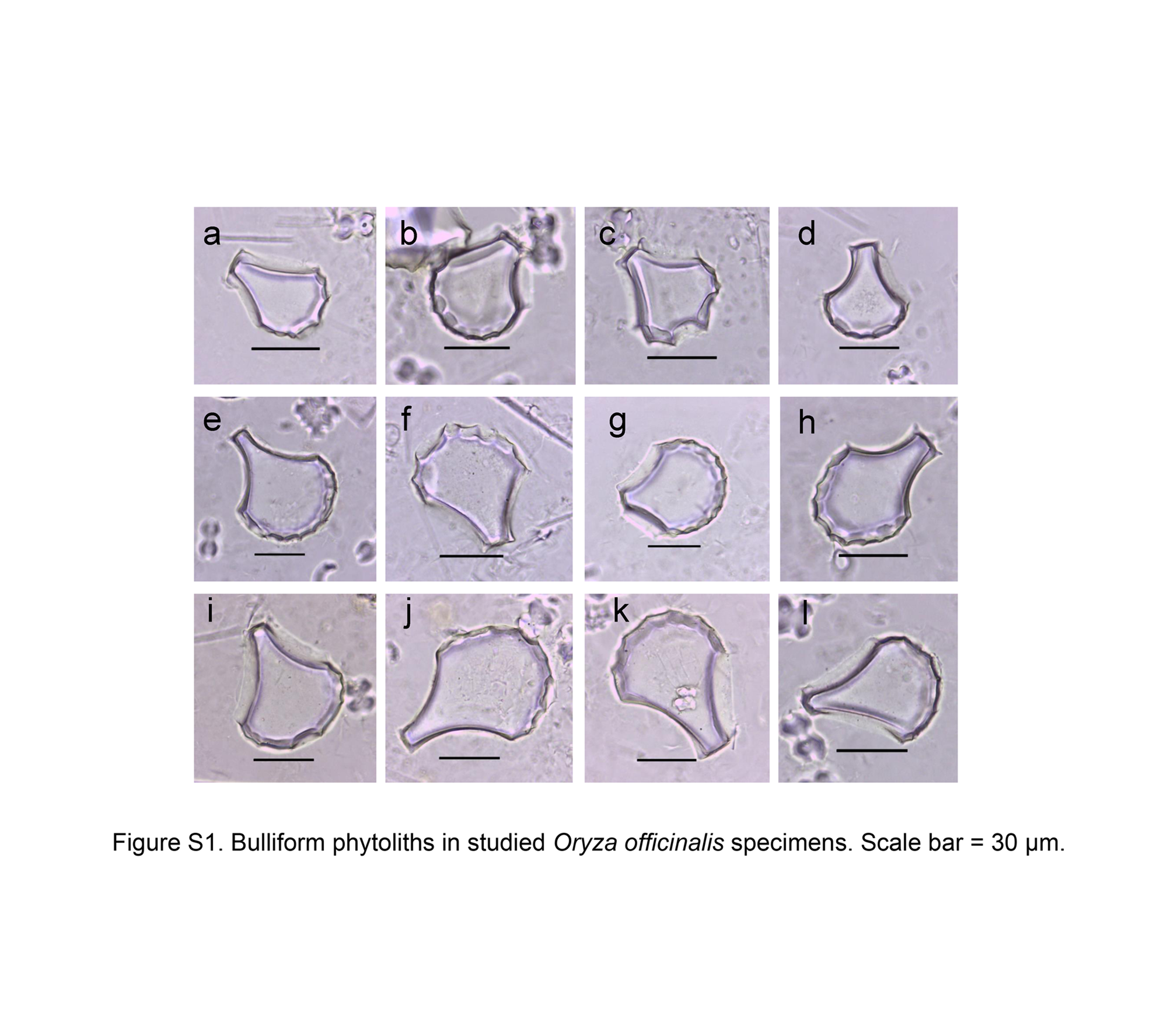

Supplement: Supplementary file 2 [file Image_1.tif]

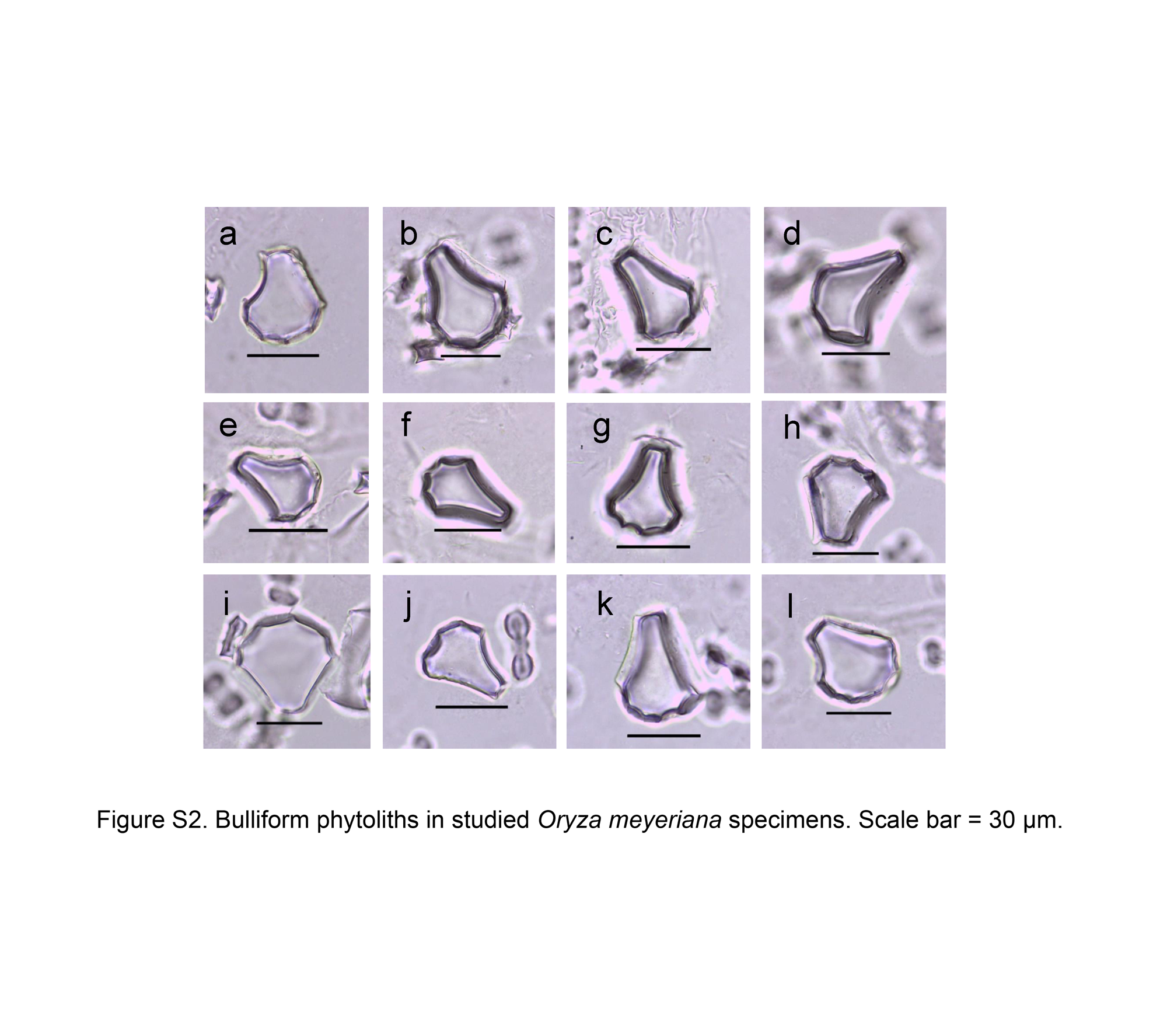

Supplement: Supplementary file 3 [file Image_2.tif]
