# Supplementary material for: Bulliform Phytolith Size of Rice and Its Correlation With Hydrothermal Environment: A Preliminary Morphological Study on Species in Southern China
Source: Front Plant Sci. 2019 Aug 22;10:1037. doi: 10.3389/fpls.2019.01037 (PMC6735168; doi:10.3389/fpls.2019.01037)
Supplement: Supplementary file 4 [file Table_1.docx]

Table S1 Structure matrix and canonical discriminant function coefficients.

|  |  | VL | HL |
| --- | --- | --- | --- |
| Structure matrix | Function 1 | 0.909 | 0.513 |
|  | Function 2 | 0.417 | 0.858 |
| Standardized coefficients | Function 1 | 1.518 | -0.738 |
|  | Function 2 | -0.908 | 1.607 |
